# Effect of Hydroxyapatite Microspheres, Amoxicillin–Hydroxyapatite and Collagen–Hydroxyapatite Composites on Human Dental Pulp-Derived Mesenchymal Stem Cells

**DOI:** 10.3390/ma14247515

**Published:** 2021-12-08

**Authors:** Yasmine Mendes Pupo, Lidiane Maria Boldrini Leite, Alexandra Cristina Senegaglia, Liziane Antunes, Jessica Mendes Nadal, Eliane Leal de Lara, Rafael Eiji Saito, Sandra Regina Masetto Antunes, William Fernandes Lacerda, Paulo Vitor Farago

**Affiliations:** 1 Department of Restorative Dentistry, Postgraduate Program in Dentistry, Federal University of Parana, Av. Prefeito Lothário Meissner 632, Jardim Botânico, Curitiba 80210-170, Brazil; yasmine.pupo@ufpr.br (Y.M.P.); williamlacerda@ufpr.br (W.F.L.); 2 Core for Cell Technology, School of Medicine, Pontifical Catholic University of Parana, Curitiba 80215-901, Brazil; lidiane.leite@pucpr.br (L.M.B.L.); alexandra.senegaglia@pucpr.br (A.C.S.); 3 Department of Chemistry, State University of Ponta Grossa, Ponta Grossa 84010-330, Brazil; prof.lizianeantunes@outlook.com (L.A.); leal.eliane@yahoo.com (E.L.d.L.); rafaeijisaito@hotmail.com (R.E.S.); sr-antunes@bol.com.br (S.R.M.A.); 4 Department of Pharmaceutical Sciences, State University of Ponta Grossa, Ponta Grossa 84010-330, Brazil; jessicabem@hotmail.com (J.M.N.); pvfarago@gmail.com (P.V.F.)

**Keywords:** bioactive scaffold materials, dentistry, cell biology, microfilled composite, molecular biology, stem cells

## Abstract

In this study, the preparation and characterization of three hydroxyapatite-based bioactive scaffolds, including hydroxyapatite microspheres (HAps), amoxicillin–hydroxyapatite composite (Amx–HAp), and collagen–hydroxyapatite composite (Col–HAp) were performed. In addition, their behavior in human dental pulp mesenchymal stem cell (hDPSC) culture was investigated. HAps were synthesized through the following methods: microwave hydrothermal, hydrothermal reactor, and precipitation, respectively. hDPSCs were obtained from samples of third molars and characterized by immunophenotypic analysis. Cells were cultured on scaffolds with osteogenic differentiation medium and maintained for 21 days. Cytotoxicity analysis and migration assay of hDPSCs were evaluated. After 21 days of induction, no differences in genes expression were observed. hDPSCs highly expressed the collagen IA and the osteonectin at the mRNA. The cytotoxicity assay using hDPSCs demonstrated that the Col–HAp group presented non-viable cells statistically lower than the control group (*p* = 0.03). In the migration assay, after 24 h HAps revealed the same migration behavior for hDPSCs observed compared to the positive control. Col–HAp also provided a statistically significant higher migration of hDPSCs than HAps (*p* = 0.02). Migration results after 48 h for HAps was intermediate from those achieved by the control groups. There was no statistical difference between the positive control and Col–HAp. Specifically, this study demonstrated that hydroxyapatite-based bioactive scaffolds, especially Col-Hap, enhanced the dynamic parameters of cell viability and cell migration capacities for hDPSCs, resulting in suitable adhesion, proliferation, and differentiation of this osteogenic lineage. These data presented are of high clinical importance and hold promise for application in therapeutic areas, because Col–HAp can be used in ridge preservation, minor bone augmentation, and periodontal regeneration. The development of novel hydroxyapatite-based bioactive scaffolds with clinical safety for bone formation from hDPSCs is an important yet challenging task both in biomaterials and cell biology.

## 1. Introduction

Development of new biomaterials or their structural change has been intensively proposed in order to enhance the final properties of novel biomedical devices [1]. Hydroxyapatite (HAp; (Ca_10_(PO_4_)_6_(OH)_2_) is thermodynamically stable in body fluid due to the fact of its crystalline state, and it has a very similar composition to mineral bone [2]. Thus, numerous techniques to synthesize hydroxyapatite-based materials at elevated temperatures (750–1000 °C) have been used in order to achieve accurate control of their structure. These procedures may be divided into two major groups: wet-chemical methods and solid-state reactions [3,4]. However, other authors have reported more a detailed classification: (1) dry methods involving solid-state and mechanochemical ones; (2) wet methods involving chemical precipitation, hydrolysis, sol-gel, hydrothermal, emulsion, and sonochemical ones; (3) high-temperature processes including combustion and pyrolysis methods; (4) synthesis based on biogenic sources; (5) combination procedures [2].

The method chosen has a remarkable influence on the morphology of the HAp obtained and can modify its crystallographic and chemical structure of powder [2]. In addition, preparation of nanosized HAp is related to a number of other problems including difficulties in controlling geometry, size and size distribution, crystallinity, stoichiometry, and the degree of particle agglomeration [2]. It is well known that the in vitro and in vivo biological and mechanical properties of HAp are strongly affected by its structural characteristics; hence, extensive efforts have been devoted to engineering HAp crystals, in particular, by developing new routes or modifications of pre-existing methods [2]. An improved microstructure with a large specific surface area could contribute to provide osteogenic spaces as compartment houses, and undifferentiated mesenchymal cells might recognize the release of Ca^2+^ and PO_4_ ^3−^ from the biological apatite layer and differentiate into osteogenic cells [5].

Two particular compounds can form composites with HAp: amoxicillin and collagen, resulting in modified HAps. Amoxicillin (α-aminohydroxybenzylpenicillin) is a semi-synthetic and broad-spectrum antibiotic, belonging to the β-lactam family, that is effective against bacterial infections [6,7]. Amoxicillin acts by inhibiting bacterial cell wall synthesis and is susceptible to beta-lactamase degradation [7]. Furthermore, the incorporation of antibiotics, such as amoxicillin, into the scaffold to provide effective mechanical properties and antibacterial activity with no cytotoxicity has been suggested as an appropriate alternative for bone infection treatment [8]. Collagen is part of the dermal structure, an original component of natural bone tissue, and plays an important role in bone regeneration, and it is considered to be a suitable biological material for tissue engineering applications [9]. It was reported that high-density collagen has been used as a material for tissue repair and regeneration [10,11], not only for its biological properties, such as low toxicity, weak antigenicity, and biocompatibility, but also due to the fact of its abundance in cartilage, tendons, and other animal structures [9]. Thus, the introduction of collagen is conducive to a good combination of scaffolds with the surrounding tissues [9].

In this sense, the preparation of modified HAps or its composites requires a careful biological investigation to guarantee its safe use. Cellular studies involving human dental pulp stem cells (hDPSCs) have been widely explored as a prior parameter to predict the safety of dental material. These hDPSCs can be easily isolated from dental medical wastes, extracted teeth, and expanded ex vivo. hDPSCs are a type of mesenchymal stem cell with the potential for cell-mediated therapy and tissue engineering applications because of their low morbidity after collection, easy surgical access, ability to be cryopreserved, ability to be recombined with many scaffolds, and their immune-privilege and anti-inflammatory abilities [12,13,14]. However, bone formation from hDPSCs requires a special structure provided by scaffolds, which should provide an appropriate environment for cellular attachment, growth, and differentiation [15]. Thus, novel hydroxyapatite-based materials need to be tested using hDPSCs in order to investigate their potential as a suitable environment for cell growth and, at the same time, provide safety for clinical use. 

In this study, the following HAp-based materials were obtained and characterized: hydroxyapatite microspheres (HAp); amoxicillin–hydroxyapatite composite (Amx–HAp); collagen–hydroxyapatite composite (Col–HAp). These scaffolds could play a critical role in attachment, survival, migration, proliferation, and differentiation. However, their effect on hDPSCs remains unknown. Therefore, we investigated the effects of HAp-based materials by in vitro cell culture-based assays on osteogenic differentiation, cytotoxicity, and migration of hDPSCs. Our null hypothesis was that these HAp-based materials, in particular Amx–HAp and Col–HAp, reduce osteogenic differentiation, cause high hDPSC death, and prevent cell migration process [16,17]. 

## 2. Materials and Methods

This work was approved by the Research Ethics Committee of the Federal University of Parana-Brazil (CAAE: 65794316.4.0000.0102). All samples were collected after the completing of an informed consent form. Three HAp-based materials were tested: hydroxyapatite nanosheet-assembled microspheres (HAps); Amx–HAp; Col–HAp. The tests were performed with hDPSCs from three different donors.

### 2.1. Synthesis and Characterization of HAp-Based Materials

#### 2.1.1. Synthesis of HAp-Based Materials

HAp, Amx–HAp, and Col–HAp were synthesized using three synthesis methods: microwave hydrothermal, reactor hydrothermal (Teflon pouches), and precipitation, respectively. 

Two aqueous solutions of 0.0835 mol/L calcium acetate (Sigma–Aldrich, ACS reagent, ≥99%) and 0.0501 mol/L ammonium dihydrogen phosphate (Sigma–Aldrich, ACS reagent, ≥99.99%) were prepared as precursors to obtain hydroxyapatite microspheres (HAp) using the microwave-hydrothermal method at a 1.67 stoichiometric ratio of Ca/P. The complexing agent citric acid monohydrate (Sigma–Aldrich, ACS reagent, ≥99.5%) was added after mixing the calcium and phosphorus precursors until a pH = 4 was achieved. Urea (Sigma–Aldrich, ACS reagent, ≥99%) was then added at a 0.016 mol/L concentration. The reaction mixture was transferred to reactors that were inserted into a microwave oven (Milestone Stard D, Sorisole, Bergamo, Italy). The microwave oven was set for a gradual heating from room temperature (20 °C) to 180 °C at 1000 W power. The synthesis time for reaching 180 °C was 5 min. The precipitate was vacuum filtered, washed twice, and oven dried. The HAps were used as a control for comparative purposes.

The Amx–HAp was prepared via the reactor-hydrothermal method using Teflon pouches [18]. Aqueous solutions of calcium acetate and ammonium dihydrogen phosphate at the same concentrations (0.0835 and 0.0501 mol/L, respectively) were used. Citric acid monohydrate was added to calcium acetate solution under magnetic stirring for 30 min to adjust pH at 4. This final solution was dripped under the ammonium dihydrogen phosphate solution, and urea at 0.016 mol/L was added. The synthesis procedure was carried out into an autoclavable reactor at 180 °C for 24 h. After drying, amoxicillin was blended by milling at a final ratio of 4.80 mg HAp:1 mg Amx. This method was performed in a high-density polypropylene flask using yttria-stabilized zirconia spheres in ethanol as a grinding agent for 3 h. After milling, Amx–HAp was oven dried for 24 h at 35 °C.

The Col–HAp was obtained using the precipitation method. This reaction was performed by adding 1.2 mol/L phosphoric acid (85% pure) into an aqueous suspension of 2.0 mol/L calcium hydroxide at a Ca/P molar ratio of 1.67 under magnetic stirring at 40 °C. The phosphoric acid dropping was controlled at 1 drop/s. The pH was then adjusted to 10.0 using ammonium hydroxide (28% pure). The precipitate was aged for 36 h, vacuum filtered, and oven dried. Collagen was also blended by ball milling at a ratio of 4.80 mg HAp:1 mg Col.

#### 2.1.2. Characterization of HAp-Based Materials

Hydroxyapatite-based materials were characterized by Fourier-transformed infrared (FTIR) spectra that were recorded from 4000 to 400 cm^−1^ on an Excalibur Bio-Rad FTS 3500 GX (Bio-Rad Laboratories, Cambridge, MA, USA). An IR spectrophotometer using KBr pellets with 32 scans and resolution of 2 cm^−1^ was used [19]. Morphological characterization was performed using field-effect emission gun scanning electron microscopy (FEG-SEM, TESCAN, model MIRA 3, Brno, Czech Republic) at various magnifications. The samples were previously deposited on polished stubs and then sputter-coated with gold. The average particle size of the biomaterials were obtained by photon correlation spectroscopy (Zetasizer Nanoseries, Malvern Instruments, Malvern, UK) after diluting each sample in ultrapure water (1:500, *v*/*v*) with no previous filtration and sonicating for 30 min. 

### 2.2. Collection and Characterization of Human Dental Pulp-Derived Mesenchymal Stem Cells (hDPSCs)

Prior to the collection of permanent teeth, the dental surgeon requested that the patient perform a buccal rinse with chlorhexidine to remove possible contaminants, guaranteeing the integrity of the material collected at the Federal University of Parana (Department of Stomatology). The tooth (3rd molar) was withdrawn from the collection flask and washed in phosphate buffer saline (PBS) (Gibco^TM^ Invitrogen, New York, NY, USA). In Petri dishes, the tooth pulp fragments were mechanically removed using endodontic files (Hedstrom Files, Kerr Manufacturing Co., Romulus, MI, USA). In the sequence, cell suspension was dissociated by collagenase type II (Gibco^TM^, Carlsbad, CA, USA) under agitation at 37 °C for 1 h, filtered (40 μm), diluted in PBS, and centrifuged. Subsequently, the pulp cells were plated in a flask with a 25 cm^2^ growth area containing 5 mL of Iscove′s modified Dulbecco′s medium (IMDM) (Gibco^TM^, Carlsbad, CA, USA) supplemented with 20% fetal bovine serum (FBS) (Gibco^TM^, Carlsbad, CA, USA), and 1% antibiotic (100 units/mL penicillin and 100 μg/mL streptomycin) (Gibco^TM^, Carlsbad, CA, USA) and were stored in an incubator at 37 °C with 5% CO_2_ tension. The culture medium was replaced three times a week. When the cultures reached approximately 80–90% confluence, enzymatic dissociation was performed using trypsin/EDTA (0.25%) (Gibco^TM^, Carlsbad, CA, USA). Inactivation of the enzyme was performed with FBS and IMDM. The cell suspension was centrifuged, the supernatant discarded, and the cells counted and plated again. All experiments were performed between the third (P3) and fifth passage (P5) of the cells.

#### Characterization of Human Dental Pulp-Derived Mesenchymal Stem Cells (hDPSCs)

Immunophenotypic analysis was performed by staining 5 × 10^5^ expanded hDPSCs. The cells were incubated with conjugated monoclonal antibodies against the following antigens: CD14 (fluorescein isothiocyanate (FITC) conjugated), CD45 (FITC conjugated), CD19 (FITC conjugated), CD90 (phycoerythrin (PE) conjugated), CD73 (PE conjugated), HLA-DR (peridinin chlorophyll protein (PerCP), CD34 (allophycocyanin (APC) conjugated), CD105 (APC conjugated), and CD29 (APC conjugated). All antibodies were from BD Pharmigen™ (San Jose, CA, USA), and they were used as suggested by the manufacturer. The incubations were performed at room temperature for 30 min. Isotype identical antibodies served as controls. After incubation, the cells were washed with PBS and fixed with PBS containing 1% paraformaldehyde (Sigma–Aldrich, São Paulo, Brazil). Samples were acquired (100,000 cells) in a BD FACSCalibur Flow Cytometer (Becton Dickinson, San Jose, CA, USA), and quantitative analyses were performed using FlowJo software v8.0.2 (Tree Star, Ashland, OR, USA).

### 2.3. In Vitro Cell Culture-Based Assays

#### 2.3.1. Experiment of Osteogenic Differentiation

Positive control of reactions was performed by inducing the differentiation of hDPSCs using an osteogenic induction medium (Differentiation Basal Medium-Osteogenic, Lonza, Walkersville, MD, USA) 37 °C with 5% CO_2_ tension and were maintained for 21 days. The culture medium was replaced three times every seven days. The differentiation was performed under the same conditions for each biomaterial tested: HAp, Amx–HAp, and Col–HAp. Osteoblastic differentiation was confirmed by mineral deposition of the culture, which was assessed by Alizarin red S (Sigma-Aldrich, São Paulo, Brazil) staining using an optical microscope (NIKON Eclipse Ni, Tokyo, Japan).

After 21 days total RNA was extracted with the TRIzol Reagent (Invitrogen, NY, USA), according to the manufacturer′s instructions. To degrade any contamination of DNA, RNA was treated with DNAse I (Qiagen, Germantown, MD, USA). Complementary DNA (cDNA) was synthesized from 1 µg of total RNA, using oligo-dT primers (USB Corporation, Cleveland, OH, USA) and a reverse transcriptase kit (ImPROm-II, Promega, Madison, WI, USA), according to the manufacturer′s instructions. Polymerase chain reaction (PCR) was carried out with 100 ng of cDNA as the template, 5 pmol of each primer, Taq polymerase and reaction mix (IBMP, Brazil). Primers used were as follows: COL1A, 5′GGCCATCCAGCTGACCTTCC3; 5′CGTGCAGCCATCGACAGTGAC3′ OSTEONECTINA′ 5′ACATCGGGCCTTGCAAATACATCC 3′; 5′ GAAGCAGCCGGCCCACTCATC 3′. We subject 20 µL of RT-PCR products to electrophoresis in a 2% agarose gel. The bands obtained were visualized by GelRed^®^ Nucleic Acid Gel Stain (Biotium, Fremont, CA, USA) and photographed under ultra-violet transillumination (UV White Darkroom, UVP Biomaging Systems, CA, USA). Glyceraldehyde-3-phosphate dehydrogenase (GAPDH) transcript was used as internal control (5′ GGCGATGCTGGCGCTGAGTAC 3′ and 5′ TGGTTCACACCCATGACGA 3′).

#### 2.3.2. Experiment of Cytotoxicity Analysis

One hundred thousand hDPSCs were plated in two T25 culture flasks and cultured in incubator at 37 °C with 5% CO_2_ tension and 95% humidity for five days with IMDM medium and 10% FBS. The differentiation was realized for each condition tested: HAp, Amx-HAp, Col–HAp, and control groups. The evaluation was performed using 7-aminoactinomycin D (7-AAD) (BD Pharmingen, San Jose, CA, USA), a fluorescent dye with high affinity for DNA that is used as a cell viability dye. Cells with compromised membranes are stained with 7-AAD, evidencing non-viable cells. Shortly after cellular dissociation, 7-AAD dye was added to the cells and then incubated for 30 min at room temperature (~22 °C). Samples were acquired in a BD FACSCalibur Flow Cytometer (Becton Dickinson, San Jose, CA, USA) and analyzed using Software FlowJo Version 8.0.2 (Tree Star, Ashland, OR, USA).

#### 2.3.3. Experiment of Migration Assay

To test whether biomaterials stimulate the chemotaxis of hDPSCs, a cell migration assay was performed using a Boyden chamber with an 8.4 mm diameter and 8 μm pore size (ThinCert™, Greiner Bio-One, Americana, Brazil) in a 24-well plate (CELLSTAR^®^, Greiner Bio-One, Americana, Brazil). The method used for this assay was adapted from Galler et al. [20]. Duplicate experiments were performed with the three hDPSCs samples. Prior to the start of the experiment, hDPSCs were cultured for four days with 1% FBS. A total of 2 × 10^5^ HDPSCS in IMDM with 1% FBS were seeded at the top of the Boyden chamber at a volume of 50 μL. After four hours, 150 μL of the same solution was added to the upper chamber, and the bottom was filled with each biomaterial in IMDM with 10% PBS, IMDM with 1% FBS (negative control), or IMDM with 20% PBS (positive control) for a final volume of 400 μL. The experiment was incubated at 37 °C and 5% CO_2_ for 24 h and 48 h. The cells in the upper chamber were carefully removed with a cotton swab. The cells that migrated to the lower chamber were washed with 70% ethanol (Labmaster, Pinhais, Brazil) and incubated with 0.2% crystal violet (Sigma–Aldrich, São Paulo, SP, Brazil) for 10 min. The excess dye was removed with distilled water and the samples were transferred to a 96-well plate (CELLSTAR^®^, Greiner Bio-One, Americana, Brazil). The assay was evaluated on a microplate reader in a 595 nm filter (Nova Analítica, São Paulo, Brazil). 

### 2.4. Statistical Analysis

The normal distribution of the migration assay was assessed by the D′Agostino and Pearson test. The data were classified as nonparametric. The comparisons between each biomaterial with the negative and positive controls as well as the biomaterials between them were analyzed with the Kruskal–Wallis test with the Dunn post-test. The results are presented as the mean absorbance ± SD. Results were considered significant at *p* < 0.05. The analyses were performed using GraphPad Prism version 5.03 for Windows (GraphPad Software, San Diego, CA, USA).

## 3. Results

### 3.1. Results of Synthesis and Characterization of HAp-Based Materials

**Figure 1:**
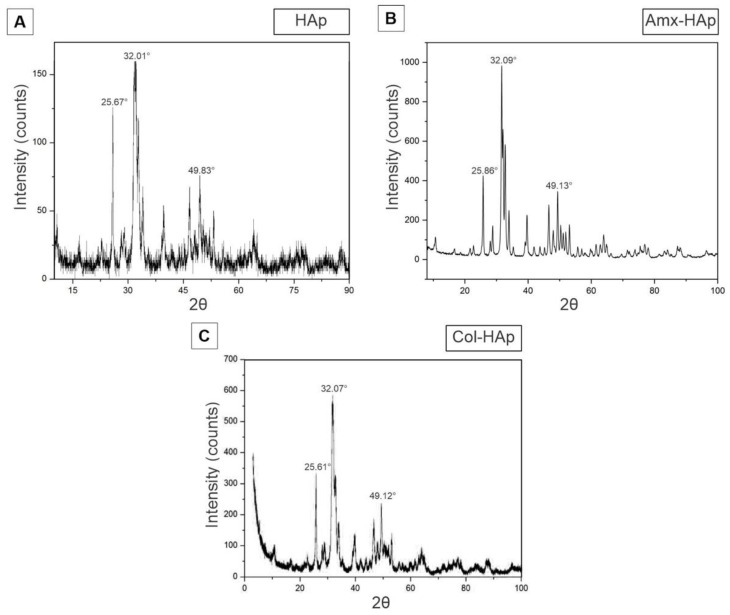
Characterization of HAp-based materials by X-ray diffractogram: (**A**) Hap, hydroxyapatite nanosheet-assembled microspheres; (**B**) Amx–Hap, amoxicillin–hydroxyapatite composite; (**C**) Col–HAp, collagen–hydroxyapatite composite.

HAp-based materials were successfully obtained using the three proposed methods, and their characterization data are depicted in Figure 1. Considering the X-ray diffraction (XRD) analysis, the XRD patterns of HAp (Figure 1A), Amx–HAp (Figure 1B), and Col–HAp (Figure 1C) presented typical crystalline peaks achieved for pure HAp (JCPDS no: 09-0432) in which the three most intense peaks were assigned at 2θ of 31.8°, 32.2°, and 32.9° and corresponded to the planes (211), (112), and (300), respectively. The hydroxyapatite microspheres (HAps) were obtained by the microwave-hydrothermal method and displayed wide peaks, which can be associated with a low degree of crystallinity due to the short crystallization time [21]. Amx–HAp showed more defined and higher intensity crystalline peaks. Col–HAp demonstrated an intermediate pattern of crystallinity. The presence of Amx and Col only led to slight shifting of the main intense crystalline peaks.

**Figure 2:**
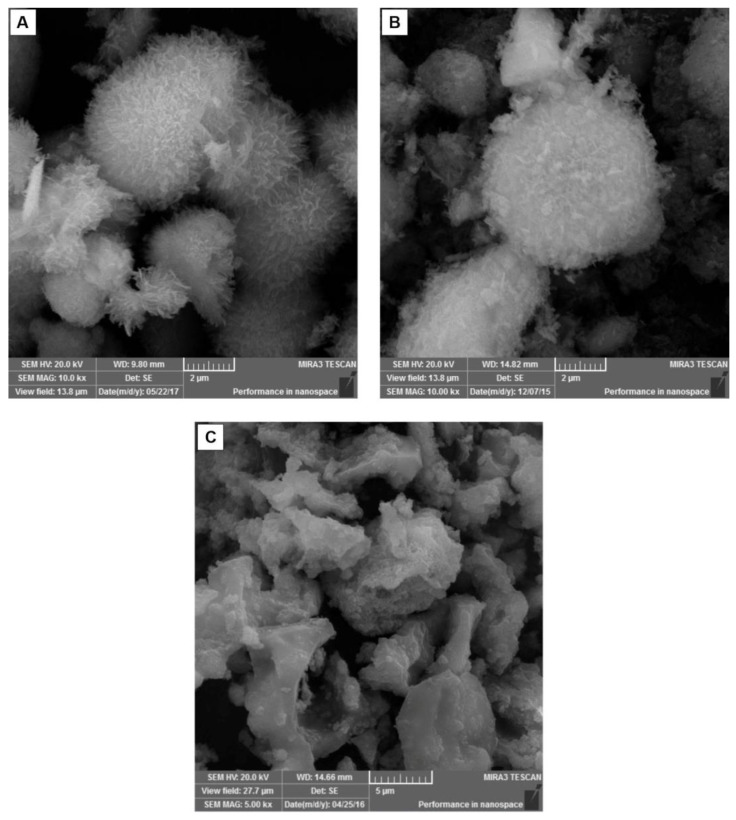
Characterization of HAp-based materials by FESEM photomicrographs: (**A**) HAps, hydroxyapatite nanosheet-assembled microspheres, 10 kx magnification; (**B**) Amx–HAp, amoxicillin–hydroxyapatite composite, 10 kx magnification; (**C**) Col–HAp, collagen–hydroxyapatite composite, 500× magnification.

The microwave-hydrothermal method provided biomaterial (HAp, Figure 2A) as nanosheet-assembled microspheres. These microparticles presented a mean particles size of 2305 ± 532 nm. The Amx–HAp composite (Figure 2B) showed similar morphological features to HAp with a mean particle size of 2743 ± 538 nm. The Col–HAp composite (Figure 2C) exhibited irregular microparticles with a smooth surface and a mean particle size of 7814 ± 653 nm. 

**Figure 3:**
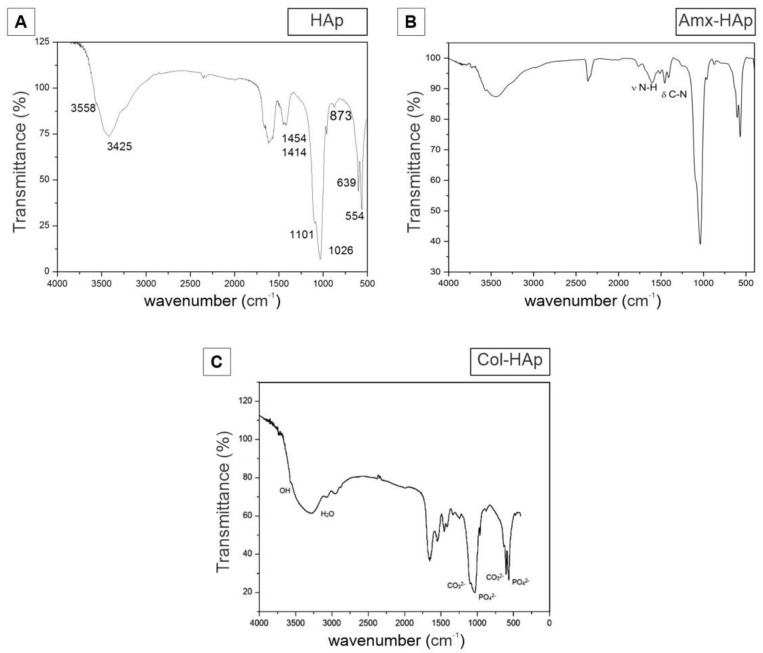
Characterization of HAp-based materials by FTIR spectra: (**A**) HAps, hydroxyapatite nanosheet-assembled microspheres; (**B**) Amx–HAp: amoxicillin–hydroxyapatite composite; (**C**) Col–HAp: collagen–hydroxyapatite composite.

HAp synthesized using the microwave-hydrothermal procedure (Figure 3A) showed broad bands at 3425 and 1620 cm^−1^ that were attributed to adsorbed water, while the sharp peak at 3558 cm^−1^ was assigned to the stretching vibration of the lattice OH− ions, and the medium sharp peak at 639 cm^−1^ was assigned to the OH deformation mode. Some typical bands for PO_4_^3−^ were observed at 554, 873, 1026, and 1101 cm^−1^. These bands confirmed that this biomaterial was consistent to HAp in accordance with the literature [22].

Three more representative bands were recorded for Amx–HAp (Figure 3B) at 1776, 1612, and 1409 cm^−1^, which were assigned to the C=O stretching of the β-lactam ring, aromatic C=C bending, and symmetric COO^–^ stretching, respectively. These assignments confirmed that this antibacterial drug was physically mixed to HAp as previously reported [23]. Col–HAp (Figure 3C) revealed additional FTIR absorption bands related to this structural protein. A signal recorded at 1655 cm^−1^ corresponded to amide I, where the peptide C=O groups contributed to stretching vibrations. Moreover, a band at 1545 cm^−1^ was assigned to the C–N stretching vibrations and N–H bending of amide II. An absorption band at 1245 cm^−1^ was related to amide III due to the stretching and binding vibrations of C–O and N–H, respectively [24]. Thus, collagen was also suitably blended to HAp as proposed.

### 3.2. Results of Collection and Characterization of Human Dental Pulp-Derived Mesenchymal Stem Cells (hDPSCs)

**Figure 4:**
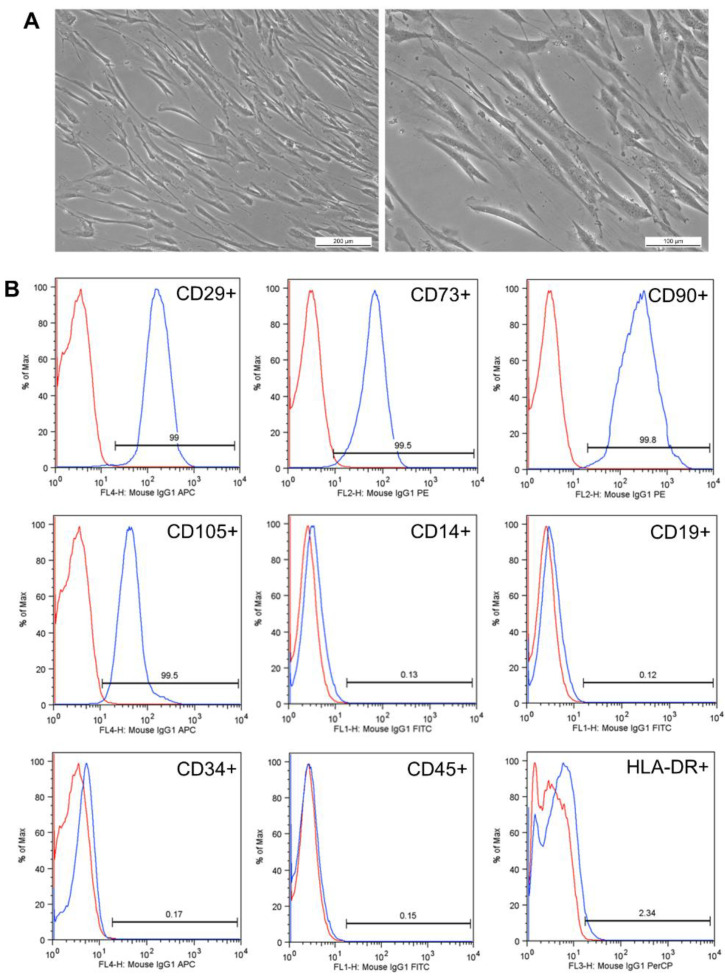
Characterization of hDPSCs. (**A**) Representation of a sample in the third passage. hDPSCs had plastic adherence and a fibroblastoid morphology. The images were magnified by 100× and 200×, respectively. (**B**) hDPSC surface markers of a study sample. In red, the isotype control is shown, and in blue, the positive markers CD29, CD73, CD90, and CD105 and the negative markers CD14, CD19, CD34, CD45, and HLA-DR are represented. The results are expressed as a percentage (%).

Dental pulp samples of permanent teeth were obtained from three patients with a mean age of 27 years. Visual observation under bright field microscopy showed a fibroblastoid morphology and capacity to adhere to plastic and, after isolation, took on average 20 days to proliferate and exceed 80% confluence (Figure 4A).

#### Results of Characterization of Human Dental Pulp-Derived Mesenchymal Stem Cells (hDPSCs)

The analysis of cell characterization showed an immunophenotypic profile compatible with the definition of mesenchymal stem cells, as suggested by Dominici et al. [25], for all three samples. The mean scores for each marker were as follow: positive markers CD29 (98.97%), CD73 (99.17%), CD90 (99.63%), and CD105 (99.27%); negative for CD14 (0.22%), CD19 (0.09%), CD34 (0.12%), CD45 (0.21%), and HLA-DR (1.5%) (Figure 4B). The cells had a mean viability of 99.55% and 0.4% of the cells were Annexin-V stained, which was indicative of apoptosis.

### 3.3. Results of Osteogenic Differentiation

**Figure 5:**
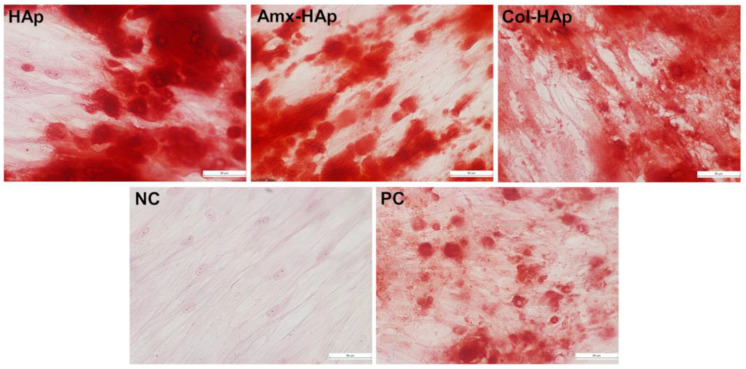
Induction of osteogenic differentiation of hDPSCs by the biomaterials tested in this study. HAps, hydroxyapatite nanosheet-assembled microspheres; Amx–HAp, amoxicillin–hydroxyapatite composite; Col–HAp, collagen–hydroxyapatite composite; NC: negative control; PC: positive control. Calcium crystals were present in the HAps, Amx–HAp, Col–HAp, and the positive control.

The ability of osteogenic differentiation was compared between the positive control and each biomaterial (HAp, Amx–HAp, and Col–HAp). The cells cultured with commercial osteogenic differentiation medium (positive control) demonstrated the presence of calcium crystals after Alizarin red S staining as well as those cultured with HAp-based materials in the study (Figure 5).

**Figure 6:**
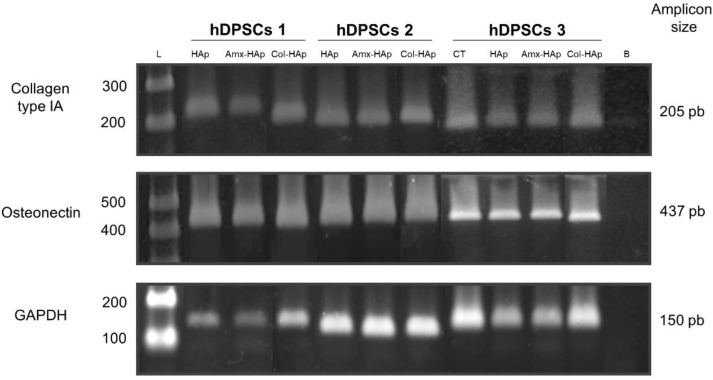
Expression of the collagen type IA and osteonectin markers of osteogenesis by PCR. Comparison between the control (hDPSCs with no osteogenic induction) and three samples of hDPSCs (submitted to six different mediums of osteogenic induction). CT, control; HAps, hydroxyapatite nanosheet-assembled microspheres; Amx–HAp, amoxicillin–hydroxyapatite composite; Col–Hap, collagen–hydroxyapatite composite; L, ladder; B, blank; GAPDH, glyceraldehyde-3-phosphate dehydrogenase, housekeeping gene; hDPSCs: dental pulp-derived mesenchymal stem cells.

No difference in gene expression was observed after 21 days of induction. hDPSCs with no induction and those under different conditions of induction highly expressed collagen IA and osteonectin at mRNA, which indicates that these genes played an important role in odontogenesis regardless of induction stimulus (Figure 6).

### 3.4. Results of Cytotoxicity Analysis

The cytotoxicity results from the cell viability assay using 7-AAD dye showed that all groups had a low mortality rate. The samples depicted the following mean ± SEM of stained non-viable cells: control (1.473 ± 0.318%), HAp (1.417 ± 0.019%), Amx–HAp (1.403 ± 0.110%), and Col–HAp (0.757 ± 0.113%). The Col–HAp group presented a number of non-viable cells statistically lower than the control group (*p* = 0.03). The other groups did not demonstrate statistical difference when compared with the control group (*p* > 0.05). 

### 3.5. Results of Migration Assay

**Figure 7:**
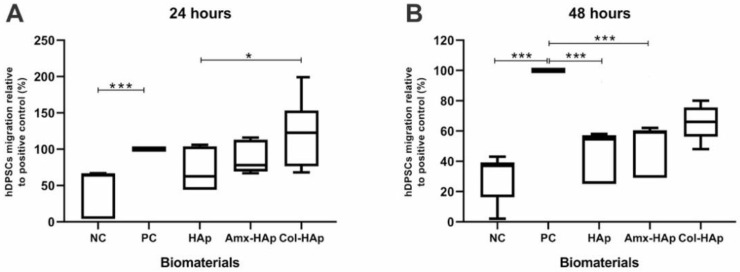
Migration assay of hDPSCs through 8 mm pore membranes toward the tested biomaterials. NC, negative control (IMDM with 1% FBS); PC, positive control (IMDM with 20% FBS); HAps, hydroxyapatite microspheres; Amx–HAp, amoxicillin–hydroxyapatite composite; Col–HAp, collagen–hydroxyapatite composite. The positive control was normalized for the 100% migration rate. Statistical analysis was performed using the Kruskal–Wallis test with the Dunn post-test; *p*-values: * *p* < 0.05 and *** *p* < 0.001. (**A**) Migration assay after 24 h. (**B**) Migration assay after 48 h.

The results obtained in the migration assay after 24 h (Figure 7A) showed that the negative control was the only group with a statistically lower migration of hDPSCs when compared with the positive group (NC vs. PC, 0.3087 ± 0.2117 vs. 0.7840 ± 0.2493 nm, *p* = 0.0008). In that sense, the biomaterials HAp, Amx–HAp, and Col–HAp revealed the same migration behavior for hDPSCs as observed with the positive control. Col–HAp also provided a statistically significant higher migration of hDPSCs than HAp (Col–HAp vs. HAp, 0.9272 ± 0.3835 vs. 0.5165 ± 0.1367 nm, *p* = 0.0248).

During the 48 h evaluation, there was a significant difference between the negative control and the positive control (NC vs. PC, 0.3857 ± 0.1747 vs. 1.498 ± 0.6080 nm, *p* < 0.0001). The migration results obtained for the biomaterials HAp, Amx–HAp, and Col–HAp were intermediate from those achieved by the control groups (Figure 7B). There was no statistical difference between the positive control and Col–HAp (*p* > 0.05).

## 4. Discussion

Studies concerning the synthesis of biomaterials, such as hydroxyapatites, with different particle sizes and forms are required for obtaining suitable scaffolds in tissue engineering for achieving both osteoinductive and dentinogenic potential. Furthermore, advanced biomaterials inspired by drugs and biological molecules can broaden prospects in terms of new technologies and variety of applications [26]. 

In recent years, various HAp-based biomaterials with different morphologies, such as nanowires [27], nanoneedles [28], nanoflowers [29], hierarchically nanostructured porous microspheres [30,31,32,33], and microflowers [34], have been synthesized. In addition, HAps are adequate candidates as drug or protein delivery carriers [35,36,37]. The biomaterials obtained showed crystalline peaks attributed to pure HAps. Thus, both Amx and Col were incorporated as non-crystalline (amorphous) materials in a composites structure. The synthetic methods for the preparation of HAp-based materials have a remarkable influence on the morphology of final bioactive scaffolds.

The microwave-hydrothermal HAp and Amx–HAp demonstrated high superficial areas, since these biomaterials were arranged as nanosheet-assembled microspheres as previously reported [38]. These 3D flower-like structures assembled with nanosheets radially oriented may affect the interaction with hDPSCs. Moreover, the Amx–HAp composite demonstrated more disordered and aggregated nanosheets than HAp, probably due to the β-lactam antibiotic′s presence and the milling effect. The Col–HAp composite exhibited irregular microparticles with a smooth surface, which resulted in a comparatively lower surface area. The FTIR spectra confirmed the main stretching and bending vibrations due to the main chemical groups of HAp, Amx, and Col. This analysis suggests that the composites were suitably obtained, since no novel chemical bonds were assigned during the biomaterials′ preparation. 

After the hDPSCs immunophenotypic characterization, this study was focused on investigating if HAp, Amx–HAp, and Col–HAp may alter the induction of osteogenic differentiation of hDPSCs through Alizarin Red S staining and immunohistochemistry analysis since this analysis can reveal the presence of calcium stores in hDPSCs extracellular matrix [39]. The biomaterials HAp, Amx–HAp, and Col–HAp did not avoid the osteogenic differentiation of hDPSCs compared to the control groups. Hence, these data evidence that these biomaterials per se are able to induce hDPSC osteogenic differentiation in vitro; nevertheless, the presence of osteogenic differentiation promoting factors accelerates and strengthens this process as resulted from the substantial augmentation of number and size of calcium content in the extracellular matrix [39]. Moreover, it has already been shown that scaffolds containing HAp improved cell proliferation and promoted the production of mineralized extracellular matrix more than that observed for the scaffold without HAps [40].

Regarding to the cytotoxicity, no HAp-based biomaterial increased the percentage of non-viable cells when compared with the control group. In addition, Col–HAp was able to preserve the cell viability significantly better than the control group. This is in line with data reported in the literature; in fact, HAp appears to have great potential for bone tissue engineering, as it showed no toxic effect on cell culture studies and also has good affinity of cellular attachment on the developed material surface. In addition, collagen is a natural component of bone tissue, where it stimulates mesenchymal stem cells to differentiate into osteoblasts initiating new bone formation [41]. Collagen also increases water retention, which facilitates cell attachment [42]. However, different methods were used for preparing the hydroxyapatite-based materials, thus leading to different morphologies, distribution size as well as surface functionalities. Hence, in addition to the collagen functionalization, these other structural aspects of each biomaterial may influence the obtained results.

Considering that DPSCs are a vital source of osteoprogenitor cells that play a fundamental role in the mechanisms related to pulp tissue plasticity [43], a migration assay was performed. The biomaterials HAp, Amx–HAp, and Col–HAp showed a similar behavior than the positive control group in the migration assay after 24 h. Col–HAp also demonstrated this result after 48 h. Migration is a key property in bone development and treatment of bone defects, since the cell chemotaxis and the invasion of cells through the extracellular matrix are responsible for ensuring the encouraging effect of a bone substitute biomaterial [44,45]. All the HAp-based biomaterials were feasible for stimulating hDPSCs migration. In this context, Col–HAp suitably acted as a chemo-attractant agent due to the fact of its ability to provide a directional hDPSC response after 24 and 48 h. 

The reported evidence remarkably demonstrates that the osteo-inductive features of the studied HAp-based biomaterials, mainly the Col–HAp scaffold, support the potential of hDPSCs inducing the formation of new bone tissue. These data suggest that these materials may represent a suitable tool for the promotion of the migration, proliferation, and differentiation of bone cells by enhancing regeneration and fracture healing.

## 5. Conclusions

HAps, Amx–HAp, and Col–HAp were successfully obtained and characterized. These biomaterials proved to ensure the osteogenic differentiation of hDPSCs, to be non-cytotoxic, and to stimulate hDPSC migration. The Col–HAp scaffold obtained by the precipitation method showed better features for the dynamic parameters of cell viability and cell migration capacities for hDPSCs.

These data present high clinical importance because Col–HAp can be used in a wide variety of therapeutic areas including ridge preservation, minor bone augmentation, and periodontal regeneration. In addition, the development of novel hydroxyapatite-based bioactive scaffolds with clinical safety for bone formation from hDPSCs is an important yet challenging task both in biomaterials and cell biology.

## Data Availability

The data presented in this study are available upon request from the corresponding author. The data are not publicly available due to the fact of ongoing studies in the field.
